# Low-Dose Steroid Therapy Is Associated with Decreased IL-12 Production in PBMCs of Severe Septic Patients

**DOI:** 10.1155/2016/1796094

**Published:** 2016-07-31

**Authors:** Huang-Pin Wu, Chi-Chung Shih, Duen-Yau Chuang, Tien-Hsing Chen

**Affiliations:** ^1^Division of Pulmonary, Critical Care and Sleep Medicine, Chang Gung Memorial Hospital, Keelung 204, Taiwan; ^2^Chang Gung University College of Medicine, Taoyuan 333, Taiwan; ^3^Department of Emergence, Chang Gung Memorial Hospital, Keelung 204, Taiwan; ^4^Department of Chemistry, National Chung-Hsing University, Taichung 402, Taiwan; ^5^Division of Cardiology, Chang Gung Memorial Hospital, Keelung 204, Taiwan

## Abstract

*Background.* Sepsis-induced immunosuppression may result in higher mortality rates in patients.* Methods*. We examined the relationship of cytokine responses from stimulated peripheral blood mononuclear cells (PBMCs) and monocyte human leukocyte antigen-DR (HLA-DR) expression (days 1 and 7) with low-dose steroid therapy in 29 septic patients. Patients were treated according to the guidelines. Thirty healthy controls were enrolled for validation.* Results*. Eighteen patients were prescribed low-dose steroids and 11 were not. Interleukin- (IL-) 12 responses in patients without low-dose steroid therapy on days 1 and 7 were higher than those with low-dose steroid therapy. Compared to day 1, IL-12 responses significantly increased on day 7 in patients without low-dose steroid therapy. After regression analysis, the change in the IL-12 response from day 7 to day 1 was found to be independently associated with the low-dose steroid therapy. There was no difference in monocyte HLA-DR expression between patients treated with and without low-dose steroid on day 1 or 7. No change in monocyte HLA-DR expression from day 7 to day 1 was observed in patients with or without low-dose steroid therapy.* Conclusion*. Decreased IL-12 response was associated with the low-dose steroid therapy in PBMCs of septic patients.

## 1. Introduction

Severe sepsis is characterized by acute release of systemic inflammatory (systemic inflammatory response syndrome [SIRS]) and anti-inflammatory mediators (compensatory anti-inflammatory response syndrome [CARS]) caused by infection [1, 2]. The cytokines associated with SIRS include interleukin- (IL-) 1*β*, IL-6, IL-12, IL-17, and tumor necrosis factor- (TNF-) *α*. The cytokines associated with CARS include IL-4, IL-10, and transforming growth factor- (TGF-) *β*1. This immune imbalance results in multiorgan dysfunctions and eventually death in patients with severe sepsis [3].

Dexamethasone inhibited lipopolysaccharides- (LPS-) stimulated release of TNF-*α*, IL-6, IL-8, and IL-10 from whole blood of septic patients [4]. Similarly, hydrocortisone decreased IL-1 and IL-6 production from* ex vivo* LPS-stimulated whole blood of septic shock patients [5]. Steroid might affect cytokine production from circulatory immune cells of septic patients.

According to the Surviving Sepsis Campaign (SSC) Guidelines for the management of severe sepsis and septic shock (2008 and 2012), patients with septic shock can be prescribed low-dose steroids if adequate fluid resuscitation and vasopressor therapy are unable to restore hemodynamic stability [6, 7]. It is different from the SSC Guidelines of 2004 version [8]. The major cause is the results of the CORTICUS study [9]. Septic shock patients treated with low-dose steroids could reverse shock at an early stage, but final mortality rate was similar to those without low-dose steroids. Low-dose steroid use may also harm patients with septic shock. Low-dose therapy was associated with an increase in adjusted hospital mortality [10]. The adjusted hospital mortality rate was significantly higher (odds ratio = 1.18, *p* < 0.001) in patients who received low-dose steroids compared with those who did not. However, a multicenter observation study found that steroid use was associated with low mortality rates in patients with severe sepsis [11]. Thus, the use of low-dose steroids in patients with severe sepsis is still controversial [12].

Serial increase in monocyte human leukocyte antigen-DR (HLA-DR) expression and IL-12 response in stimulated peripheral blood mononuclear cells (PBMCs) are associated with higher survival rate in patients with severe sepsis [13]. Thus, we analyzed our study database to explore the relationship of low-dose steroid treatment with HLA-DR expression and cytokine responses in patients with severe sepsis by repeated detections.

## 2. Materials and Methods

This study is a post hoc analysis using our previously published study database [13].

### 2.1. Participants and Definitions

From July 2008 to June 2009, 35 patients who were admitted to a 20-bed intensive care unit (ICU) in a regional teaching referral hospital for severe sepsis were enrolled in this study. Six nonsurvivors died within 7 days and they all received low-dose steroid therapy. The data of these six patients was excluded because of lack of repeated cytokine response results for analysis. The SIRS was defined as two or more of the following criteria: (1) body temperature > 38°C or <36°C; (2) respiratory rate > 20 breaths/min; (3) heart rate > 90 beats/min; and (4) white blood count > 12000/*μ*L or <4000/*μ*L or >10% bands. Sepsis was defined as SIRS according to a confirmed infectious etiology. For validating experimental findings, 22 men and 8 women visiting our health evaluation center for examinations were enrolled as healthy controls with mean age of 60.8 ± 1.9 years old.

Severe sepsis was defined according to the consensus criteria of sepsis with one or more organ dysfunctions such as shock, respiratory failure, acute renal failure, jaundice, and thrombocytopenia [14, 15]. Septic shock was defined as sepsis-induced hypotension unresponsive to fluid resuscitation within 24 hr after admission to ICU. Respiratory failure was defined as ventilation dysfunction requiring invasive ventilator support. Acute renal failure was defined as a rapid increase in creatinine levels (>0.5 mg/dL). Jaundice was defined as hyperbilirubinemia (total bilirubin > 2 mg/dL), whereas thrombocytopenia was defined as a platelet count of <150,000/*μ*L. Disease severity was assessed by the Acute Physiology and Chronic Health Evaluation (APACHE) II score [16].

Standard treatment according to guidelines was provided to all patients [6, 8]. A course of low-dose steroid therapy could be prescribed in septic shock patient with 7 days of intravenous hydrocortisone 50 mg every 6 hours if shock developed within 24 hr after admission to ICU. The Institutional Review Board at Chang Gung Memorial Hospital approved our previous study (96-1465B) and the patients' close family members provided informed consent. Patients who survived longer than 28 days after ICU admission were defined as survivors.

### 2.2. PBMCs Preparation

Whole blood (10 mL) was obtained from each patient at 08:30 a.m., within 48 h of admission to ICU, and immediately mixed with heparin. The day of first blood sampling was defined as day 1. A second blood sample was obtained on day 7. PBMCs were isolated via differential centrifugation over Ficoll-Plaque (Amersham Biosciences, Uppsala, Sweden) from 8 mL of residual whole blood within 2 h of collection.

### 2.3. Monocyte HLA-DR Measurement by Flow Cytometry

2.5 × 10^5^ of PBMCs were suspended in 50 *μ*L of phosphate-buffered saline (PBS) and incubated in the dark for 15 min at room temperature with 20 *μ*L of HLA-DR_PerCP_, CD11b_PE_, and CD14_FITC_ antibodies (Becton Dickinson, CA, USA). Then, the cells were resuspended in 500 *μ*L of PBS. The monocytes were detected by a three-color flow cytofluorimeter (Beckman Coulter, CA, USA) with positive controls for CD11b_PE_ and CD14_FITC_. Monocyte HLA-DR measurements were expressed as percentages of HLA-DR-positive monocytes and as means of fluorescence intensities (MFI) in relation to the entire monocyte population, thus reflecting the HLA-DR density per cell. Flow cytometry analysis was performed using Kaluza software V1.1 (Beckman Coulter, CA, USA). Setting gates were based on the internal negative population. The figure of analysis strategy for monocyte HLA-DR expression was presented in our previously published paper [13].

### 2.4. Cell Culture

5 × 10^5^ PBMCs were plated in two wells of a flat-bottomed 24-well plate (Nunclon, Aarhus, Denmark) in 1 mL of sterile RPMI 1640 tissue culture medium containing 5% heat-inactivated bovine serum, 1 mM of L-glutamine (Gibco, Grand Island, USA), and 1 mM sodium pyruvate. The cells in first well were not stimulated or treated. The cells in second well were stimulated with 1 pg/*μ*L of LPS (Sigma, Missouri, USA). The plate was incubated at 37°C in 5% carbon dioxide for 24 h. Supernatants of the culture wells were sampled and stored at −80°C until use.

### 2.5. Measurement of Cytokine Levels

Cytokine levels of supernatants were measured with a human enzyme-linked immunosorbent assay (ELISA) kit, according to the manufacturer's instructions. The ELISA kit of IL-10 was manufactured by Pierce Biotechnology, Illinois, USA. The IL-6, TGF-*β*1, and IL-17 were purchased from R&D Systems, Inc., Minnesota, USA. The IL-12, TNF-*α*, and IL-1*β* were purchased from Becton Dickinson, CA, USA. Cytokine responses were defined as the difference in supernatant levels with and without LPS stimulation. Negative responses were set as 0 pg/mL. Changes in cytokine responses were defined as the difference in cytokine response on day 7 minus the cytokine response on day 1.

### 2.6. Statistical Analysis

Statistical analysis was performed using the Statistical Package for the Social Sciences (SPSS) software V17.0 for Windows (SPSS Inc., Illinois, USA). Differences in continuous variables between two groups were analyzed using the Mann-Whitney test, whereas differences in categorical variables were analyzed using the chi-square test or Fisher's exact test. Differences in continuous variables in the same subjects were analyzed using the Wilcoxon signed-rank test. Generalized linear model analysis was used to determine the association between clinical characteristics and cytokine response. A *p* value < 0.05 was considered statistically significant.

## 3. Results

Of the final 29 enrolled subjects with severe sepsis, 18 patients were prescribed low-dose steroids and 11 were not (Table 1). In the low-dose steroid group, 12 patients survived for 28 days and six died (Table 2). All 11 patients that did not receive low-dose steroid therapy survived. The APACHE II score in low-dose steroid group was higher than no-steroid group, although the statistical analysis did not show difference. There were no significant differences in age, gender, histories, infection sources, and initial appropriateness of antibiotics between groups with and without low-dose steroid therapy. Patients administered low-dose steroid therapy displayed higher percentages of septic shock (94% versus 36%), compared with no low-dose steroid group. The rates of gastrointestinal bleeding, acute renal failure, thrombocytopenia, jaundice, bacteremia, and mortality were similar between the two groups.

### 3.1. Cytokine Responses between Patients with and without Low-Dose Steroid Therapy

TNF-*α* response in the control group was significantly higher than in both patient groups (Table 3). However, IL-1*β* response in the control group was significantly lower than in both patient groups. IL-12 and TNF-*α* responses on days 1 and 7 in steroid group were significantly lower than in no-steroid group. There were no differences in IL-1*β*, IL-6, IL-10, IL-17, and TGF-*β*1 responses on days 1 and 7 between the two patient groups.

### 3.2. Change of Cytokine Responses from Day 7 to Day 1 in Subjects

**Figure 1:**
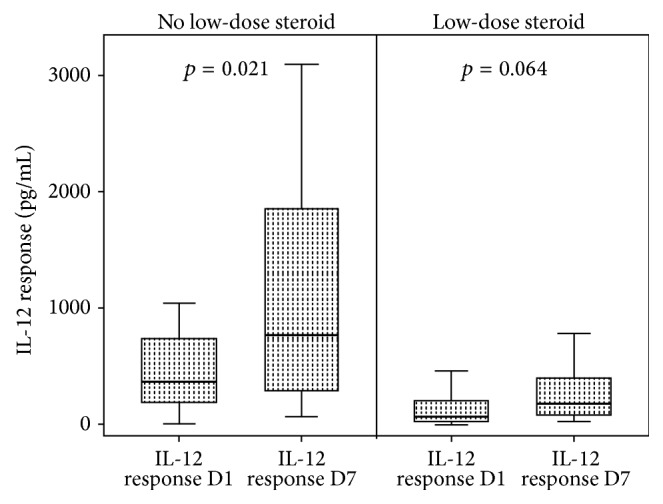
Interleukin- (IL-) 12 responses significantly recover in no low-dose steroid group. The boxplots show median of IL-12 response in day 1 and day 7 in groups with and without low-dose steroid treatment.

IL-1*β* and TGF-*β*1 responses in patients administered low-dose steroid therapy decreased from day 7 to day 1 (Table 3). Compared to day 1, there were no changes in IL-6, IL-10, IL-12, IL-17, and TNF-*α* responses on day 7 in patients who received low-dose steroid therapy. IL-12 response in patients without low-dose steroid treatment significantly increased from day 7 to day 1 (Figure 1). Similarly, there were no changes in IL-1*β*, IL-6, IL-10, IL-17, TGF-*β*1, and TNF-*α* production in patients treated without low-dose steroid therapy from day 7 to day 1.

In survivors with severe sepsis, IL-12 response increased and IL-6 response decreased significantly from day 7 to day 1. There were no changes in IL-1*β*, IL-10, IL-17, TGF-*β*1, and TNF-*α* production from day 7 to day 1 in survivors. All cytokine responses detected did not change from day 7 to day 1 in nonsurvivors (Table 4).

### 3.3. Association between the Change in Cytokine Responses and Low-Dose Steroid Therapy

Table 5 shows regression analysis results demonstrating the relationship between changes in IL-1*β* levels and low-dose steroid therapy and other clinical characteristics. APACHE II score levels were independently and positively associated with changes in IL-1*β* response (*B* = 3.110). Patients who were severely ill produced more IL-1*β* in PBMCs after 6 days compared with those who had milder illness. However, low-dose steroid therapy was not independently associated with the change in IL-1*β* levels. Low-dose steroid therapy did not influence IL-1*β* production in PBMCs of severe septic patients.

Low-dose steroid therapy was also independently and negatively linked to the increase in IL-12 after regression analysis (*B* = −750.743; Table 6). IL-12 recovery from day 7 to day 1 was lower in patients with low-dose steroid therapy. The presence of septic shock did not independently correlate with the change in IL-12 levels. Sex was independently related to the variation in IL-12 response. IL-12 recovery from day 7 to day 1 was high in male patients (*B* = 447.838).

Although TGF-*β*1 response in patients receiving low-dose steroidtherapy decreased from day 7 to day 1, the regression analysis did not find independent factors associated with the change in TGF-*β*1 levels (Table 7).

### 3.4. HLA-DR Expression with or without Low-Dose Steroid Therapy on Days 1 and 7

**Figure 2:**
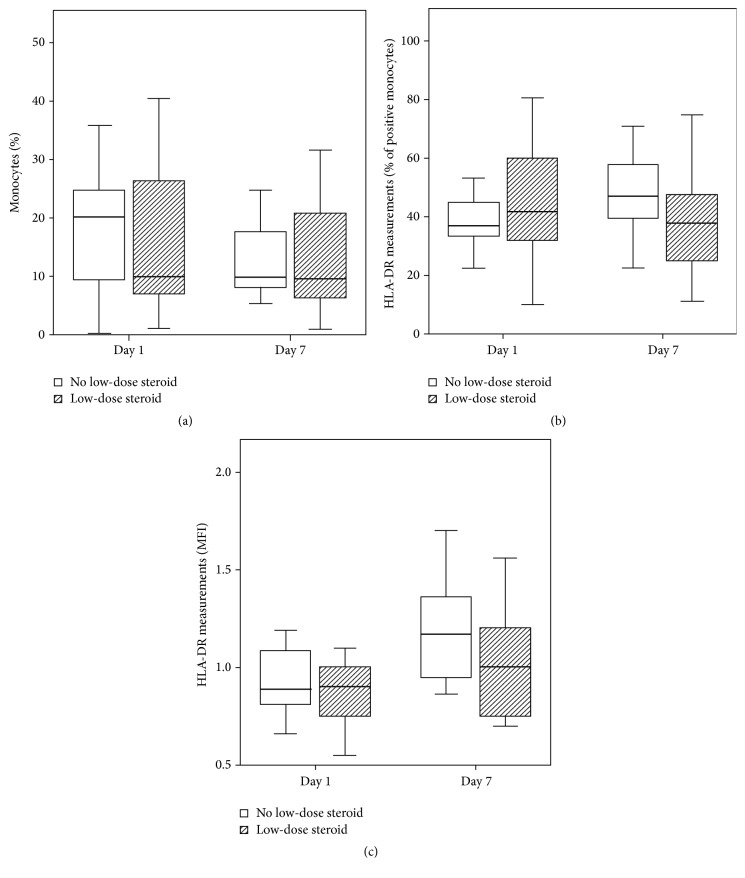
No change of HLA-DR expression between groups of no low-dose steroid and low-dose steroid. Boxplots of (a) monocyte percentage, (b) positive HLA-DR percentage in monocytes, and (c) MFI of HLA-DR are shown in patients treated with or without low-dose steroid therapy on days 1 and 7. HLA= human leukocyte antigen; MFI= means of fluorescence intensities.

There was no difference in monocyte percentage, positive HLA-DR percentage in monocytes, and MFI of HLA-DR between patients treated with or without low-dose steroids on day 1 or 7 (Figure 2). In addition, there was no change in monocyte percentage, positive HLA-DR percentage in monocytes, and MFI of HLA-DR in patients treated with or without low-dose steroids from day 7 to day 1.

## 4. Discussion

Previous studies often used* in vitro* cultures to test the effectiveness of different doses of steroids [4] or repeatedly measured plasma levels between the patients receiving or not receiving steroids at specific times [5]. These studies have a common fault; they indirectly detect the function or response of immune cells after a course of low-dose steroid therapy. Since circulating cytokines can be produced by immune and nonimmune cells, such as smooth muscle cells and endothelial cells [17], circulating cytokine levels only reflect a relatively broad response.

In this study, we demonstrated that a complete course therapy with low-dose steroid was associated with decreased IL-12 production in PBMCs from patients with severe sepsis. In contrast to our results, a 6-day crossover study with serial detecting plasma cytokine levels in patients with septic shock reported that the inhibitory effect of hydrocortisone infusion did not decrease plasma IL-12 level [18]. The first possible reason for this result is that only 3 days of hydrocortisone infusion was administered to each group; therefore, the effect of low-dose steroid did not have a chance to develop. Second, Ficoll density gradient centrifugation for monocytes isolation resulted in lower cell function than by positive selection by magnetic microbeads [19]. The function of IL-12 production from PBMCs in this study might be influenced by Ficoll solution. Other reasons may be that there is increased IL-12 production in dendritic or human B-lymphoblastoid cells, resulting in similar amounts of circulating IL-12.

In this study, we did not find an association between IL-6 response and low-dose steroid therapy. This was similar to a study by Oppert et al. They found that IL-6 production in LPS-stimulated diluted whole blood transiently fell from day 1 to 3 and returned after day 5 in a low-dose hydrocortisone therapy group [5]. In terms of circulating cytokine levels, there were no significant differences in plasma IL-6 levels between treatment groups of continuous 6 h infusion of endotoxin + hydrocortisone or endotoxin + saline in an animal study [20]. Furthermore, a double-blind, randomized, placebo-controlled study showed similar results [21]. Two doses of hydrocortisone (100 mg per 8 h) were administered after bilateral total knee replacement in the study group. IL-6 levels were 40% lower in the study group after 10 h but returned to levels similar to that of the control group at 24 h. However, other studies have reported different results. Stress dose of hydrocortisone infusion in patients with septic shock significantly decreased plasma IL-6 levels on day 5 between the steroid and placebo groups [22]. Hydrocortisone infusion reduced plasma IL-6 levels in a 6-day crossover study in patients with septic shock [18]. Generally, low-dose steroid therapy may influence plasma IL-6 levels but not IL-6 response in PBMCs, based on current evidence.

The effect of steroid therapy on IL-10 production is controversial. Stimulated monocyte production of IL-10 was enhanced at low concentrations with glucocorticosteroid therapy [23]. This effect was not observed in our study. IL-10 production in stimulated PBMCs did not differ between patients receiving or not receiving low-dose steroid therapy on day 1 or 7. Even after 6 days of therapy, IL-10 response did not change in the low-dose steroid group. Moreover, dexamethasone inhibited LPS-stimulated release of IL-10 from diluted whole blood of septic patients in a dose-dependent manner [4]. After* in vivo* administration of high or low amounts of cortisol, high cortisol therapy further increased LPS-induced IL-10 expression in isolated monocytes from healthy participants, whereas low cortisol therapy decreased Il-10 expression [24]. More studies are needed to determine the role of low-dose steroid therapy on IL-10 production in patients with severe sepsis.

Endogenous cortisol is one of the main anti-inflammatory mediators induced by our central nervous system during severe sepsis. It has been described that steroids have downregulating effects on monocyte HLA-DR expression [25–27]. High endogenous cortisol levels observed in septic shock patients may play a role in the loss of monocyte HLA-DR expression via its effect on HLA-DR transcription. However, monocyte HLA-DR expression seems not to be influenced by low-dose steroid therapy. In this study, there was no change in monocyte percentage, positive HLA-DR percentage in monocytes, and MFI of HLA-DR in patients with low-dose steroid therapy from day 1 to 7. Similar results have been reported by Keh et al. [18]. Patients with low-dose hydrocortisone therapy showed unchanged monocyte HLA-DR expression. More studies are needed to confirm our and Keh et al.'s results.

Cortisol can influence the immune system and is crucial for the host for defense against pathogens. The endocrine system may be also influenced by the immune system. During inflammation, cytokines mediate a high glucocorticosteroid output with regulation from the neuroendocrine to the immune-endocrine system [28]. As a result, high levels of adrenal glucocorticosteroid are vital in preventing an uncontrolled inflammatory response to cytokines.

The IL-12 response from PBMCs was restored in patients who survived severe sepsis [13]. Severe septic survivors produced more IL-12 from LPS-stimulated PBMCs than nonsurvivors [29]. The main immunological function of IL-12 is to enhance native T lymphocyte differentiation to type 1 help T (Th1) cells. Th1 cells secrete interferon-*γ* that regulates macrophage and natural killer (NK) cell activation, stimulates immunoglobulin secretion by B cells, and enhances Th1 cell differentiation. In this work, low-dose steroid therapy resulted in decreased IL-12 production. This might explain why low-dose therapy was associated with an increase in adjusted hospital mortality in SSC database [10]. Decreased IL-12 response in patients receiving low-dose steroid therapy may depress a protective effect by decreased cellular immunity and phagocytic functions.

There were two limitations in this work. First, the percentage of septic shock was different between steroid and no-steroid groups. The reason why steroid group had higher percentage of shock is that patients with septic shock are appropriate for steroid treatment according to guidelines. In septic shock group, not in no-shock group, patients with low-dose steroid had significantly lower change of IL-12 response than those without low-dose steroid. Although generalized linear model analysis was used to exclude the confounding effect of shock, there was not a directly statistical analysis to demonstrate low-dose steroid affected IL-12 response recovery in patients without septic shock. Second, patients with low-dose steroid treatment have received steroid when their blood was drawn on day 1. Although the time from onset of steroid treatment to blood sampling was short and not more than 48 hr, cytokine responses and HLA-DR expression might be influenced.

## 5. Conclusions

IL-12 response observed in PBMCs increased from day 7 to day 1 in severe septic patients. We demonstrated that a course of low-dose steroid therapy influenced IL-12 production from* in vitro* LPS-stimulated PBMCs of severe septic patients. Low-dose steroid therapy was associated with less increase of IL-12 response. There was no correlation between low-dose steroid therapy and monocyte HLA-DR expression.

## Figures and Tables

**Table 1:** Clinical characteristics of severe septic patients with and without low-dose steroid use (percentage, mean ± standard error mean).

	No-low-dose steroid (*n* = 11)	Low-dose steroid (*n* = 18)	*p* value
Age (years old)	72.3 ± 6.4	72.1 ± 2.2	0.132
Male	64	72	0.694
APACHE II score	24.5 ± 2.4	29.2 ± 1.5	0.142
History			
COPD	36	22	0.433
Heart failure	9	11	1.000
Hypertension	45	22	0.237
Diabetes mellitus	0	22	0.268
End stage renal disease	0	6	1.000
Liver cirrhosis	9	11	1.000
Infection source			0.595
Pneumonia	91	78	
Urinary tract infection	9	16	
Biliary tract infection	0	6	
Initial antibiotics for pathogens			0.367
Sensitive	36	61	
Resistant	36	28	
No pathogen isolated	27	11	

APACHE = acute physiology and chronic health evaluation; COPD = chronic obstructive pulmonary disease.

**Table 2:** Adverse event and outcome (number, percentage).

	No-low-dose steroid (*n* = 11)	Low-dose steroid (*n* = 18)
Event		
Gastrointestinal bleeding	2 (18)	2 (11)
Acute renal failure	4 (36)	11 (61)
Shock	4 (36)	17 (94)^*∗*^
Thrombocytopenia	3 (27)	5 (28)
Jaundice	0 (0)	1 (6)
Bacteremia	1 (9)	2 (11)
Mortality	0 (0)	6 (33)^†^

^*∗*^ *p* = 0.001 compared to no-low-dose steroid group, by Fisher's exact test.

^†^ *p* = 0.058 compared to no-low-dose steroid group, by Fisher's exact test.

**Table 3:** Cytokine responses (pg/mL, mean ± standard error mean) on days 1 and 7 in no-low-dose steroid, low-dose steroid, and control groups.

	No-low-dose steroid (*n* = 11)	Low-dose steroid (*n* = 18)	All patients (*n* = 29)	Controls (*n* = 30)
Day 1				
IL-1*β*	182.0 ± 18.6	200.0 ± 5.6	193.1 ± 7.8	110.8 ± 8.3^†^
IL-6	375.0 ± 40.2	416.2 ± 13.8	400.6 ± 17.5	385.4 ± 31.7
IL-10	340.4 ± 84.6	333.1 ± 74.1	335.8 ± 55.1	305.5 ± 29.4
IL-12	514.1 ± 151.8	141.3 ± 39.5^*∗*^	282.7 ± 69.9	266.6 ± 50.5
IL-17	4.7 ± 4.3	2.6 ± 1.3	3.4 ± 1.8	1.0 ± 0.5
TGF-*β*1	26.3 ± 15.3	92.5 ± 28.7	67.4 ± 19.5	36.0 ± 9.9
TNF-*α*	404.9 ± 39.5	279.8 ± 41.2^*∗*^	327.2 ± 31.4	540.8 ± 34.4^†^

Day 7				
IL-1*β*	189.8 ± 5.8	191.7 ± 5.7^‡^	191.9 ± 4.1	
IL-6	356.4 ± 35.2	410.3 ± 12.7	389.9 ± 15.9^‡^	
IL-10	339.3 ± 58.5	237.4 ± 41.0	276.0 ± 34.4	
IL-12	1145.7 ± 337.5^‡^	269.5 ± 57.7^*∗*^	601.9 ± 152.1^‡^	
IL-17	0.8 ± 0.4	4.6 ± 1.9	3.2 ± 1.2	
TGF-*β*1	25.3 ± 16.2	28.2 ± 11.9^‡^	27.1 ± 9.4^‡^	
TNF-*α*	434.7 ± 14.3	367.1 ± 24.5^*∗*^	392.8 ± 17.1	

IL = interleukin; TGF = transforming growth factor; and TNF = tumor necrosis factor.

^*∗*^ *p* < 0.05 compared to no-low-dose steroid group, by Mann-Whitney test.

^†^ *p* < 0.05 compared to all patients with severe sepsis, by Mann-Whitney test.

^‡^ *p* < 0.05 compared within the same group on day 1, by Wilcoxon signed-rank test.

**Table 4:** Cytokine responses (pg/mL, mean ± standard error mean) on days 1 and 7 in survivors, nonsurvivors, and control groups.

	Survivors (*n* = 23)	Nonsurvivors (*n* = 6)	All patients (*n* = 29)	Controls (*n* = 30)
Day 1				
IL-1*β*	193.2 ± 9.2	192.9 ± 15.7	193.1 ± 7.8	110.8 ± 8.3^*∗*^
IL-6	407.0 ± 21.4	376.2 ± 19.5	400.6 ± 17.5	385.4 ± 31.7
IL-10	298.1 ± 59.4	480.5 ± 132.0	335.8 ± 55.1	305.5 ± 29.4
IL-12	325.8 ± 84.1	117.6 ± 77.0	282.7 ± 69.9	266.6 ± 50.5
IL-17	3.5 ± 2.2	2.8 ± 2.3	3.4 ± 1.8	1.0 ± 0.5
TGF-*β*1	49.3 ± 17.8	136.8 ± 60.8	67.4 ± 19.5	36.0 ± 9.9
TNF-*α*	348.6 ± 35.4	245.1 ± 61.8	327.2 ± 31.4	540.8 ± 34.4^*∗*^

Day 7				
IL-1*β*	192.0 ± 3.5	187.2 ± 15.6	191.9 ± 4.1	
IL-6	394.1 ± 19.3^†^	373.5 ± 21.8	389.9 ± 15.9^†^	
IL-10	273.5 ± 39.6	285.8 ± 74.7	276.0 ± 34.4	
IL-12	696.0 ± 185.7^†^	241.1 ± 108.2	601.9 ± 152.1^†^	
IL-17	2.7 ± 1.1	4.8 ± 4.6	3.2 ± 1.2	
TGF-*β*1	27.8 ± 10.7	24.5 ± 21.9	27.1 ± 9.4^†^	
TNF-*α*	403.9 ± 15.2	349.9 ± 59.5	392.8 ± 17.1	

IL = interleukin; TGF = transforming growth factor; and TNF = tumor necrosis factor.

^*∗*^ *p* < 0.05 compared to all patients with severe sepsis, by Mann-Whitney test.

^†^ *p* < 0.05 compared within the same group on day 1, by Wilcoxon signed-rank test.

**Table 5:** Generalized linear model analysis for the change in IL-1*β* response to identify independent factors among clinical characteristics and steroid use.

	*B*	Change in IL-1*β* response or (rho)^*∗*^	*p* value
Age	−0.689	0.068	0.154
Sex			0.775
Male	−4.358	−8.6, −94.5–94.1	
Female	0^†^	−13.1, −23.5–153.5	
APACHE II	3.110	0.292	0.006
Septic shock			0.083
Yes	−34.395	−10.8, −94.5–94.1	
No	0^†^	−3.8, −28.0–153.5	
Low-dose steroid			0.472
Yes	−13.113	−10.3, −94.5–94.1	
No	0^†^	2.2, −28.0–153.5	
Mortality			0.659
Yes	7.850	−8.5, −94.5–94.1	
No	0^†^	−9.9, −38.4–153.5	

IL = interleukin; APACHE = Acute Physiology and Chronic Health Evaluation.

^*∗*^Data are presented as the median and range (in pg/mL) for categorical variables or the Spearman rank correlation coefficient (rho) value for continuous variables.

^†^0 is set as a reference.

**Table 6:** Generalized linear model analysis for the change in IL-12 response to identify independent factors among clinical characteristics and steroid use.

	*B*	Change in IL-12 response or (rho)^*∗*^	*p* value
Age	−2.369	0.015	0.727
Sex			0.036
Male	447.838	253.725, −432.0–2059.9	
Female	0^†^	58.3, −813.8–1023.4	
APACHE II	−5.300	−0.316	0.095
Septic shock			0.083
Yes	482.418	176.4, −432.0–2059.9	
No	0^†^	241.6, −813.8–1928.9	
Low-dose steroid			0.003
Yes	−750.743	87.6, −432.0–679.7	
No	0^†^	352.3, −813.8–2059.9	
Mortality			0.574
Yes	−140.572	49.3, −432.0–679.7	
No	0^†^	224.6, −813.8–2059.9	

IL = interleukin; APACHE = Acute Physiology and Chronic Health Evaluation.

^*∗*^Data are presented as the median and range (in pg/mL) for categorical variables or the Spearman rank correlation coefficient (rho) value for continuous variables.

^†^0 is set as a reference.

**Table 7:** Generalized linear model analysis for the change in TGF-*β*1 response to identify independent factors among clinical characteristics and steroid use.

	*B*	Change in TGF-*β*1 response or (rho)^*∗*^	*p* value
Age	0.296	0.039	0.789
Sex			0.351
Male	−32.608	−13.4, −253.9–173.7	
Female	0^†^	0.0, −133.6–0.0	
APACHE II	−5.110	−0.282	0.051
Septic shock			0.350
Yes	42.598	0.0, −253.9–173.7	
No	0^†^	0.0, −155.8–66.8	
Low-dose steroid			0.388
Yes	−36.088	−13.4, −253.9–66.8	
No	0^†^	0.0, −155.8–173.7	
Mortality			0.066
Yes	−75.079	−96.5, −253.8–13.4	
No	0^†^	0.0, −229.0–173.7	

TGF = transforming growth factor; APACHE = Acute Physiology and Chronic Health Evaluation.

^*∗*^Data are presented as the median and range (in pg/mL) for categorical variables or the Spearman rank correlation coefficient (rho) value for continuous variables.

^†^0 is set as a reference.

## References

[1] Oberholzer A., Oberholzer C., Moldawer L. L. (2001). Sepsis syndromes: understanding the role of innate and acquired immunity. *Shock*.

[2] Reddy R. C., Chen G. H., Tekchandani P. K., Standiford T. J. (2001). Sepsis-induced immunosuppression: from bad to worse. *Immunologic Research*.

[3] Hotchkiss R. S., Karl I. E. (2003). The pathophysiology and treatment of sepsis. *The New England Journal of Medicine*.

[4] Giamarellos-Bourboulis E. J., Dimopoulou I., Kotanidou A. (2010). Εx-vivo effect of dexamethasone on cytokine production from whole blood of septic patients: correlation with disease severity. *Cytokine*.

[5] Oppert M., Schindler R., Husung C. (2005). Low-dose hydrocortisone improves shock reversal and reduces cytokine levels in early hyperdynamic septic shock. *Critical Care Medicine*.

[6] Dellinger R. P., Levy M. M., Carlet J. M. (2008). Surviving Sepsis Campaign: international guidelines for management of severe sepsis and septic shock: 2008. *Critical Care Medicine*.

[7] Dellinger R. P., Levy M. M., Rhodes A. (2013). Surviving sepsis campaign: international guidelines for management of severe sepsis and septic shock: 2012. *Critical Care Medicine*.

[8] Dellinger R. P., Carlet J. M., Masur H. (2004). Surviving Sepsis Campaign guidelines for management of severe sepsis and septic shock. *Critical Care Medicine*.

[9] Sprung C. L., Annane D., Keh D. (2008). Hydrocortisone therapy for patients with septic shock. *The New England Journal of Medicine*.

[10] Casserly B., Gerlach H., Phillips G. S. (2012). Low-dose steroids in adult septic shock: results of the Surviving Sepsis Campaign. *Intensive Care Medicine*.

[11] Miller R. R., Dong L., Nelson N. C. (2013). Multicenter implementation of a severe sepsis and septic shock treatment bundle. *American Journal of Respiratory and Critical Care Medicine*.

[12] Patel G. P., Balk R. A. (2012). Systemic steroids in severe sepsis and septic shock. *American Journal of Respiratory and Critical Care Medicine*.

[13] Wu H.-P., Shih C.-C., Lin C.-Y., Hua C.-C., Chuang D.-Y. (2011). Serial increase of IL-12 response and human leukocyte antigen-DR expression in severe sepsis survivors. *Critical Care*.

[14] Russell J. A. (2006). Management of sepsis. *New England Journal of Medicine*.

[15] Levy M. M., Fink M. P., Marshall J. C. (2003). 2001 SCCM/ESICM/ACCP/ATS/SIS international sepsis definitions conference. *Critical Care Medicine*.

[16] Knaus W. A., Draper E. A., Wagner D. P., Zimmerman J. E. (1985). APACHE II: a severity of disease classification system. *Critical Care Medicine*.

[17] Sprague A. H., Khalil R. A. (2009). Inflammatory cytokines in vascular dysfunction and vascular disease. *Biochemical Pharmacology*.

[18] Keh D., Boehnke T., Weber-Cartens S. (2003). Immunologic and hemodynamic effects of ‘low-dose’ hydrocortisone in septic shock: a double-blind, randomized, placebo-controlled, crossover study. *American Journal of Respiratory and Critical Care Medicine*.

[19] Zhou L., Somasundaram R., Nederhof R. F. (2012). Impact of human granulocyte and monocyte isolation procedures on functional studies. *Clinical and Vaccine Immunology*.

[20] Söderberg E., Lipcsey M., Sjölin J., Larsson A., Eriksson M. B. (2012). Counteraction of early circulatory derangement by administration of low dose steroid treatment at the onset of established endotoxemic shock is not directly mediated by TNF-*α* and IL-6. *Steroids*.

[21] Jules-Elysee K. M., Lipnitsky J. Y., Patel N. (2011). Use of low-dose steroids in decreasing cytokine release during bilateral total knee replacement. *Regional Anesthesia and Pain Medicine*.

[22] Briegel J., Jochum M., Gippner-Steppert C., Thiel M. (2001). Immunomodulation in septic shock: hydrocortisone differentially regulates cytokine responses. *Journal of the American Society of Nephrology*.

[23] Hodge S., Hodge G., Flower R., Han P. (1999). Methyl-prednisolone up-regulates monocyte interleukin-10 production in stimulated whole blood. *Scandinavian Journal of Immunology*.

[24] Yeager M. P., Pioli P. A., Wardwell K. (2008). In vivo exposure to high or low cortisol has biphasic effects on inflammatory response pathways of human monocytes. *Anesthesia and Analgesia*.

[25] Asadullah K., Woiciechowsky C., Döcke W. D. (1995). Very low monocytic HLA-DR expression indicates high risk of infection—immunomonitoring for patients after neurosurgery and patients during high dose steroid therapy. *European Journal of Emergency Medicine*.

[26] Haveman J. W., van den Berg A. P., van den Berk J. M. M. (1999). Low HLA-DR expression on peripheral blood monocytes predicts bacterial sepsis after liver transplantation: relation with prednisolone intake. *Transplant Infectious Disease*.

[27] Tulzo Y. L., Pangault C., Amiot L. (2004). Monocyte human leukocyte antigen-DR transcriptional downregulation by cortisol during septic shock. *American Journal of Respiratory and Critical Care Medicine*.

[28] Bornstein S. R., Rutkowski H., Vrezas I. (2004). Cytokines and steroidogenesis. *Molecular and Cellular Endocrinology*.

[29] Stanilova S. A., Karakolev Z. T., Dimov G. S. (2005). High interleukin 12 and low interleukin 10 production after in vitro stimulation detected in sepsis survivors. *Intensive Care Medicine*.

